# Associated factors with vaccine hesitancy in mothers of children up to two years old in a Brazilian city

**DOI:** 10.1371/journal.pgph.0002026

**Published:** 2023-06-08

**Authors:** Érica Marvila Garcia, Evelyn Lima de Souza, Fernanda Penido Matozinhos, Tércia Moreira Ribeiro da Silva, Eliseu Alves Waldman, Ana Paula Sayuri Sato

**Affiliations:** 1 School of Public Health, University of Sao Paulo, Sao Paulo, Sao Paulo, Brazil; 2 Nursing School, Federal University of Minas Gerais, Belo Horizonte, Minas Gerais, Brazil; Christian Medical College Vellore, INDIA

## Abstract

This study aims to evaluate maternal vaccine hesitancy and its associated factors. This is a cross-sectional study of a probabilistic sample of 450 mothers of children born in 2015, living in a Brazilian city, and who was, at the time of data collection, more than two years old. We used the tool proposed by the World Health Organization (10-item Vaccine Hesitancy Scale). To assess its structure, we performed, exploratory and confirmatory factor analyses. We performed linear regression models to evaluate the factors associated with vaccine hesitancy. The factor analysis showed two components for the vaccine hesitancy scale: lack of confidence in vaccines and risk perception of vaccines. High family income was associated with lower vaccine hesitancy (greater confidence in vaccines and lower risk perception of vaccines), while the presence of other children, regardless of birth order, in the family was associated with lower confidence in vaccines. A good rapport with health professionals, willingness to wait for the vaccination and the getting vaccinated through campaigns were associated with greater confidence in vaccines. The deliberate delay or decision not to vaccinate their children and previous experience with adverse reactions to the vaccine were associated with lower confidence in vaccines and greater risk perception of vaccines. Health care providers, especially nurses, play a relevant role to address vaccine hesitancy, guiding vaccination through a trustworthy rapport.

## Introduction

The Brazilian National Immunization Program (NIP) is one of the most thorough immunization programs in the world, and it is renowned for its collective and individual strategies that ensured high vaccination coverages for almost all immunobiologicals for decades, allowing the progressive reduction of incidence and death rates for vaccine-preventable diseases, such as measles, polio and pertussis [1–5]. However, in 2019, seven vaccines aimed at immunizing children showed a reduction in the doses applied when compared to the previous year, amongst them, vaccine coverage for the 3rd dose of Diphtheria tetanus toxoid and pertussis (DTP), with 96.6% in 2011, but 68.43% in 2021, the poliomyelitis vaccine with 100% in 2011, while that in 2021 it was 71%, and the 1st dose of the measles vaccine with 100% coverage in 2011, however in 2021, decreased to 74.9% [6–10].

This trend in Brazil directly agrees with the impression that there has been a recent reduction in vaccine coverage in other countries [6]. In addition, the increase in the number of cases of some vaccine-preventable diseases, hitherto controlled, such as the reappearance of measles in 2018 suggests the presence of pockets of susceptible people [11].

The recent national reduction of childhood vaccination coverages associated with the development of pockets of susceptible individuals in certain areas [6,12] put herd immunity at risk and increase the chances of circulation of vaccine-preventable diseases that were hitherto controlled or eradicated [6,13–15]. Included as a strategic priority goal in the 2030 Global Immunization Agenda, high and equitable immunization coverage requires investigation of the factors and barriers that impede its accomplishment [16]. Although investigating the reasons for vaccine refusal and vaccination delay is a recommended strategic pillar by WHO for best practices in vaccination programs management, NIP is limited to vaccination coverage and dropout rate surveillance, which are insufficient indicators to identify reasons associated with vaccine hesitancy.

Acknowledging the factors that interfere in family adherence to the vaccination schedule or in population access to vaccination is fundamental to direct and evaluate vaccination programs efforts, it allows the identification of low vaccination coverage groups and the development of strategies that aim to reduce inequalities. Among these factors, studies point out that individual and familial aspects, living contexts and vaccination facilities characteristics may influence children vaccination status [14,17–21].

Vaccine Hesitancy (VH) is a major challenge for public health experts worldwide [22,23]. In 2020, a study carried out by Figueiredo et al. (2020) [22] in 149 countries, provided multiyear global-level estimates of vaccine confidence, exploring trends in confidence and the global determinants of uptake including socioeconomic determinants and sources of trust.

Canadian and European studies pointed out maternal influence in the decision to vaccinate children, highlighting those negative experiences related to vaccination, whether personal, familial or from an acquaintance, as well as a remembrance of adverse reactions to vaccines, contributed to distrust in vaccination and influenced mothers in the decision to vaccinate their children [24,25]. Trusting a pediatrician or other influential persons was fundamental to maternal decision-making to vaccinate children [25]. However, researches that investigate Canadian and European populations may not present the singularities, sociodemographic profile and social vulnerabilities commonly identified in Brazilian and in other low-and middle-income countries that may influence the decision to vaccinate children, which makes necessary the investigation of Brazilian mothers’ hesitancy to vaccination.

A study by Lane et al., using data from the WHO/UNICEF-2015–2017 joint report form, over three years, showed that vaccine hesitancy was common and reported by 90% of countries. The main reasons mentioned were related to concerns about the risks and benefits of the vaccine, lack of knowledge and barriers related to religion, culture, gender or socioeconomic factors [26].

In Brazil, few studies so far have addressed the refusal or voluntary delay of vaccines. Besides, as in other countries, there are few studies produced to better understand the causes and impact of vaccine hesitancy in the Brazilian population, making evident the need to broaden the discussion of this topic in Brazil [4,26–31].

Therefore, the objective of the present study is to evaluate factors associated with vaccine hesitancy in mothers of children up to two years old and if these factors can influence maternal decision to vaccinate children.

## Materials and methods

### Ethics statement

The project was approved by the Ethics Research Committee, as recommended in Resolution No. 466 of 2012—National Health Council for Scientific Research in Human Beings (CAAE: 20721819.0.0000.5421). Only the mothers who signed the informed consent forms were interviewed and included in the study. Data confidentiality, and its use purely for scientific purposes were guaranteed.

This is a cross-sectional study with data from a household vaccine survey of a probabilistic sample consisting in 450 mothers, whose children were born in 2015, living in the Brazilian medium-sized city, Araraquara (SP).

Located in the central region of São Paulo state, one of the most developed and socio-economically forward regions of Brazil, Araraquara has a computerized system to account for vaccine doses that are offered to the population, at no charge, by the city’s Primary Care facilities.

The inclusion criteria for mothers with children to participate in this vaccine hesitancy survey included were: (i) mothers whose children were born and living in Araraquara, (ii) mothers whose children were born in 2015 and who were, at the time of data collection, more than two years old (iii) mothers whose children were registered in electronic immunization registry (EIR), (iv) mothers that answered the questionnaire during the face-to-face interview, and (v) mothers who presented the child’s vaccination record. The exclusion criteria were: (i) death before reaching 24 months of age, (ii) children who moved to another municipality before reaching 24 months of age, and (iii) institutionalized children.

The stratified probabilistic sample was extracted by a drawing without replacing the record on an electronic immunization system. To obtain a representative sample, the city was divided into geographical units, formed by a grouping of census sectors [32]. In this way, each of the 15 geographical units of Araraquara represented a stratum. The number of interviews was proportional to the number of children born in 2015 and residents in each area.

To calculate the minimum sample size, a confidence coefficient was considered, whose value was 1.96 for an alpha of 0.05; outcome frequency of 40% [33]; and a maximum error in the absolute value of 0.05 [34]. Thus, a minimum sample size (= [1.96^2^x 0.4 x 0.6] / 0.05^2^ = 369) was obtained.

The final population for the random sample was 3,054 children, of who 450 children were drawn, considering an increase of 20% of the calculated minimum sample size.

Data collection was carried out by a trained and supervised team (10 field interviewers and 4 supervisors), from August 15 to October 30, 2018, through a face-to-face interview at the residence, scheduled by the interviewer, via telephone contact, with a duration average of 60 minutes. For the application of the questionnaire, the interviewers were trained in a 10-hour course, which covered theory and practice, to achieve quality control of information through the standardization of the approach, interview and application of the questionnaire. Five mothers participated in a pre-test to adapt the instrument and, later, 20 mothers from the city of Araraquara participated in the pilot study. The data from the mothers that participated in the pilot study did not compose the analysis.

The 10-item Vaccine Hesitancy Scale developed by the Strategic Advisory Group of Experts Working Group (SAGE-WG) from WHO, in which each item is evaluated on a 5-point Likert scale (1 = strongly disagree; 2 = disagree; 3 = neither agree nor do I disagree; 4 = agree; 5 = strongly agree), was used to assess vaccine hesitancy in mothers of children up to two years old. The tool was translated to Portuguese and validated for this study (adapted from LARSON et al., 2015 [35]; OPEL et al., 2011[36]).

Besides the variables present in the Vaccine Hesitancy Scale, variables sociodemographic, behavioral aspects, aspects related to health services and aspects related to vaccination were collected, were chosen on the basis of previous studies (Table 1).

**Table 1:** Variables sociodemographic, behavioral aspects, aspects related to health services and aspects related to vaccination included in the study.

Category	Variable
Socioeconomic and demographic characteristics of the mother and family of the child	Mother’s age; Mother’s skin/color; Marital status; Mother’s education; Total family income; Other Children; Mother’s religion;
Prenatal, delivery and puerperium characteristics of the child’s mother	Total of prenatal consultations; Guidance by health professionals during prenatal care or after delivery about the child’s vaccination; Child’s birth weight (kg); Child’s gender; The frequency of child’s attendance in daycare/school during the first two years of life; Exclusive breastfeeding duration; Child’s hospitalization in the first two years of life
Access, use and rapport with health services, child’s vaccination characteristics	Mother’s rapport with health unit professionals (assessed by the mother’s self-report as: excellent, good, reasonable, bad or indifferent); Vaccination center; Adverse reaction to any vaccine; Maximum willingness to wait to apply a vaccine; Campaign vaccination consistency; Deliberate delay or decision not to vaccinate

To assess the structure of the Vaccine Hesitancy Scale and identify the highest factor loadings of each subscale, exploratory factor analysis and confirmatory factor analysis were performed, for this reason, the sample was randomly divided into two. In the first half, it was analyzed by exploratory factor analysis, where each item was free to load on each factor. The factors were extracted using the Varimax rotation. The second half was used to validate the resulting latent structure, using confirmatory factor analysis, where the item loaded only on the factors designated by the exploratory analysis [37]. Besides, Cronbach’s alpha was used to estimate internal consistency.

The analysis of factors associated with maternal vaccine hesitancy was performed using simple and multiple linear regressions, considering the score generated for each subscale from the vaccine hesitancy in mothers of children up to two years old as the dependent variable [38]. The variables that presented p<0.20 in the univariate analysis were chosen for the adjusted model, being included through the forward strategy, while for the permanence in the final model, a significance level of 5% was assumed. The data were analyzed using the Stata14 software.

## Results

**Fig 1:**
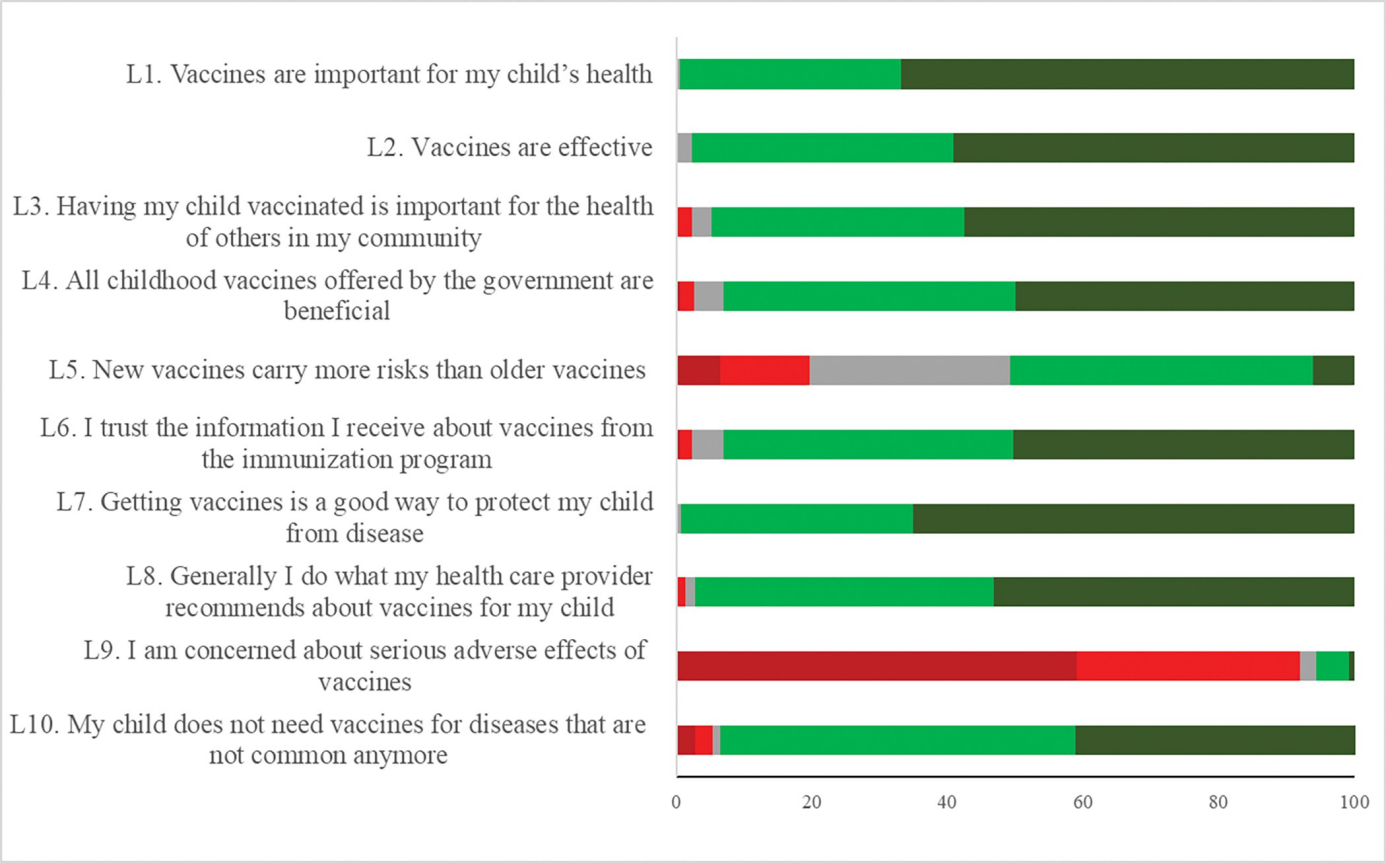
Distribution of responses on vaccine hesitancy in mothers of children up to two years old, according to behavior, negative (dark red and red), neutral (gray) and positive (dark green and green), (n = 388).

Fig 1 shows the distribution of responses related to the vaccine hesitancy scale: the red shades represent negative behavior, the gray, neutral, and the green shades, positive. A large proportion of mothers showed positive behavior towards the items: L1—Vaccines are important for my child’s health (99.5%); L2—Vaccines are effective (97.7%); L3—Having my child vaccinated is important for the health of others in my community (94.9%); L4—All childhood vaccines offered by the government are beneficial (93.0%); L6—I trust the information I receive about vaccines from the vaccination program (93.1%); L7—Getting vaccines is a good way to protect my child from disease (99.3%) and; L8—Generally I do what my health care provider recommends about vaccines for my child (97.2%). On the other hand, some mothers showed negative attitudes concerning items L5—New vaccines carry more risks than older vaccines (50.8%); L9—I am concerned about the serious adverse effects of vaccines (92.0%), and; L10—My child does not need vaccines for diseases that are not common anymore (93.5%).

The Cronbach’s alpha was 80.1%, indicating good internal consistency of the instrument. Table 2 presents the results of the exploratory and confirmatory factor analysis of the vaccine hesitancy scale items. It was considered that item 10 (L10) did not correspond to any of the factors. For the others, two factors were identified with eigenvalues of 3.90 for factor one (lack of confidence: when the mother did not trust the benefits of the vaccine) and 1.21 for factor two (risks perception: when the mother had the perception that the vaccine is a risk to the child’s life).

**Table 2:** Parameters of exploratory and confirmatory factor analysis from vaccine hesitancy in mothers of children up to two years old, (n = 388).

	Exploratory factor analysis		Confirmatory factor analysis	
	Lack of confidence	Risks perception	Lack of confidence	Risks perception
L1. Vaccines are important for my child’s health.	0.813	-0.434	0.855	-
L2. Vaccines are effective.	0.805	0.047	0.867	-
L3. Having my child vaccinated is important for the health of others in my community.	0.740	0.099	0.680	-
L4. All childhood vaccines offered by the government are beneficial.	0.622	0.301	0.658	-
L5. New vaccines carry more risks than older vaccines.	0.088	0.806	-	0.785
L6. I trust the information I receive about vaccines from the immunization program.	0.630	0.250	0.541	-
L7. Getting vaccines is a good way to protect my child from disease.	0.862	-0.063	0.860	-
L8. Generally, I do what my health care provider recommends about vaccines for my child.	0.674	0.047	0.683	-
L9. I am concerned about serious adverse effects of vaccines.	-0.236	0.607	-	0.785

Tables 3–5 show the factor analysis associated with vaccine hesitancy for both components: “lack of confidence” and “risk perception” of vaccines.

**Table 3:** Parameters of the unadjusted and adjusted linear regression models to study factors associated with lack of confidence and risk perception from vaccine hesitancy in mothers of children up to two years old, according to socioeconomic and demographic characteristics of the mother and family of the child (n = 388).

	Factor 1: Lack of confidence					Factor 2: Risk perception				
	Mean (SD)	Unadjusted model		Adjusted model		Mean (SD)	Unadjusted model		Adjusted model	
		β	P-value	β	P-value		β	P-value	β	P-value
**Mother’s age**										
<30 years	1.57 (0.04)	Ref.				3.62 (0.07)	Ref.			
30 to 35 years	1.44 (0.04)	-0.12	0.04	-	-	3.61 (0.06)	-0.01	0.89	-	-
>35 years	1.44 (0,04)	-0.12	0.03	-	-	3.48 (0.06)	-0.14	0.10	-	-
**Mother’s skin/color**										
White	1.42 (0.03)	Ref.				3.55 (0.04)	Ref.			
Not white	1.55 (0.04)	0.13	0.01	-	-	3.60 (0.06)	0.05	0.48	-	-
**Marital status**										
Without partner	1.60 (0.05)	Ref.				3.70 (0.09)	Ref.			
With partner	1.45 (0.02)	-0.15	0.01	-	-	3.54 (0.04)	-0.17	0.07	-	-
**Mother’s education**										
<8 years	1.61 (0.07)	Ref.				3.68 (0.11)	Ref.			
8 to 11 years	1.52 (0.03)	-0.09	0.29	-	-	2.59 (0.05)	-0.09	0.51	-	-
>11 years	1.35 (0.04)	-0.26	<0.01	-	-	3.49 (0.06)	-0.19	0.18	-	-
**Total family income**										
<2 MW*	1.59 (0.03)	Ref.				3.64 (0.05)	Ref.			
2 to 5 MW	1.41 (0.04)	-0.18	<0.01	-0.16	<0.01	3.51 (0.06)	-0.13	0.09	-0.17	0.03
6 to 8 MW	1.41 (0.09)	-0.18	0.06	-0.08	0.37	3.34 (0.10)	-0.30	0.04	-0.31	0.03
>8 MW	1.23 (0.08)	-0.36	<0.01	-0.32	<0.01	3.54 (0.16)	-0.10	0.52	-0.13	0.37
**Other children**										
None	1.38 (0.03)	Ref.				3.62 (0.06)	Ref.			
1 child	1.47 (0.04)	0.08	0.12	0.09	0.07	3.49 (0.05)	-0.13	0.11	-	-
≥2 children	1.62 (0.05)	0.24	<0.01	0.21	<0.01	3.63 (0.07)	0.01	0.94	-	-
**Mother’s religion**										
Without religion/ atheist	1.49 (0.09)	Ref.				3.52 (0.15)	Ref.			
Protestant	1.58 (0.04)	0.09	0.40	-	-	3.55 (0.06)	0.03	0.86	-	-
Catholic	1.40 (0.03)	-0.09	0.41	-	-	3.58 (0.05)	0.06	0.71	-	-
Umbanda/candomblé	1.43 (0.21)	-0.06	0.76	-	-	3.71 (0.29)	0.19	0.53	-	-
Spiritist	1.42 (0.12)	-0.07	0.62	-	-	3.54 (0.14)	0.02	0.92	-	-
Other religions	1.45 (0.11)	-0.04	0.77	-	-	3.56 (0.21)	0.04	0.87	-	-

*MW = Minimum Wages.

Note: SD = standard deviation; β = regression coefficient; Ref. = Reference category.

**Table 4:** Parameters of linear regression models unadjusted and adjusted for the factors related to lack of confidence and risk perception from vaccine hesitancy in mothers of children up to two years old, according to the prenatal, delivery and puerperium characteristics of the child’s mother.

	Factor 1: Lack of confidence			Factor 2: Risk perception		
	Mean (SD)	Unadjusted model		Mean (SD)	Unadjusted model	
		β	P-value		β	P-value
**Total of prenatal consultations**						
< 6 consultations	1.50 (0.10)	Ref.		3.78 (0.18)	Ref.	
≥ 6 consultations	1.47 (0.02)	-0.03	0.81	3.54 (0.04)	-0.24	0.17
**Guidance by health professionals during prenatal care or after delivery about the child’s vaccination**						
No	1.54 (0.05)	Ref.		3.54 (0.09)	Ref.	
Yes	1.46 (0.03)	-0.08	0.19	3.57 (0.04)	0.03	0.74
**Child’s birth weight (kg)**						
<2500	1.41 (0.06)	Ref.		3.59 (0.11)	Ref.	
≥2500	1.48 (0.02)	0.07	0.34	3.56 (0.04)	-0.03	0.79
**Child’s gender**						
Female	1.47 (0.03)	Ref.		3.57 (0.05)	Ref.	
Male	1.48 (0.03)	0.02	0.73	3.56 (0.05)	-0.01	0.93
**Child’s attendance at day care/school**						
No	1.50 (0.03)	Ref.		3.63 (0.05)	Ref.	
Yes	1.46 (0.03)	-0.04	0.39	3.52 (0.05)	-0.11	0.11
**Exclusive breastfeeding duration**						
<6 months	1.46 (0.03)	Ref.		3.59 (0.05)	Ref.	
≥6 months	1.49 (0.04)	0.03	0.47	3.54 (0.05)	-0.05	0.53
**Child’s hospitalization in the first two years of life**						
No	1.48 (0.02)	Ref.		3.58 (0.04)	Ref.	
Yes	1.47 (0.07)	-0.01	0.90	3.45 (0.11)	-0.13	0.25

Note: SD = standard deviation; β = regression coefficient; Ref. = Reference category.

**Table 5:** Parameters of linear regression models unadjusted and adjusted for the factors associated with lack of confidence and risk perception from vaccine hesitancy in mothers of children up to two years old, according to characteristics of access, use and rapport with health services, child’s vaccination characteristics.

	Factor 1: Lack of confidence					Factor 2: Risk perception				
	Mean (SD)	Unadjusted model		Adjusted model		Mean (SD)	Unadjusted model		Adjusted model	
		β	P-value	β	P-value		β	P-value	β	P-value
**Mother’s rapport with health unit professionals**										
Bad	1.69 (0.45)	Ref.				3.88 (0.76)	Ref.			
Reasonable	1.61 (0.46)	-0.08	0.52	-0.15	0.20	3.56 (0.65)	-0.32	0.10	-0.35	0.06
Good	1.45 (0.45)	-0.24	0.02	-0.22	0.02	3.55 (0.70)	-0.33	0.03	-0.34	0.03
**Vaccination location**										
Public health unit	1.51 (0.03)	Ref.				3.56 (0.04)	Ref.			
Private clinic	1.21 (0.05)	-0.30	<0.01	-	-	3.56 (0.11)	-0.01	0.96	-	-
**Adverse reaction to some vaccine**										
No	1.49 (0.03)	Ref.				3.49 (0.04)	Ref.			
Yes	1.42 (0.05)	-0.08	0.15	-	-	3.82 (0.07)	0.33	<0.01	0.33	<0.01
**Maximum time willing to wait to apply a vaccine**										
Less than 30 minutes	1.56 (0.03)	Ref.				3.56 (0.04)	Ref.			
From 30 to 60 minutes	1.43 (0.05)	-0.13	0.02	-0.16	<0.01	3.58 (0.07)	0.02	0.85	-	-
More than 60 minutes	1.29 (0.04)	-0.27	<0.01	-0.27	<0.01	3.59 (0.09)	0.02	0.78	-	-
Do not know		-	-	-	-		-	-	-	-
**Campaign vaccination habit**										
No	1.91 (0.26)	Ref.				3.32 (0.17)	Ref.			
Yes	1.46 (0.02)	-0.44	<0.01	-0.54	<0.01	3.58 (0.04)	0.26	0.23	-	-
**Deliberately delay or decision not to vaccinate**										
No	1.46 (0.02)	Ref.				3.53 (0.04)	Ref.			
Yes	1.61 (0.08)	0.15	0.03	0.17	0.01	3.85 (0.10)	0.32	<0.01	0.23	0.03

Note: SD = standard deviation; β = regression coefficient; Ref. = Reference category.

High family income was associated with lower lack of confidence in vaccines (income of 2 to 5 minimum wages (MW): β of -0.16 and p-value of <0.01; or above 8 times the monthly minimum wages: β of -0.32 and p-value of <0.01), and lower risk perception of vaccines (income of 2 to 5 minimum wages (MW): β of -0.17 and p-value of 0.03; or income of 6 to 8 times the monthly minimum wages: β of -0.31 and p-value of 0.03). While the presence of other children, regardless of birth order, in the family (β of 0.21 and p-value of <0.01) was associated with lower confidence in vaccines.

A good rapport with health professionals was associated with lower lack of confidence in vaccines (β of -0.22 and p-value of < 0.02) and lower risk perception of vaccines (β of -0.34 and p-value of 0.03). While willingness to wait for the vaccine application (From 30 to 60 minutes: β of -0.16 and p-value of <0.01; More than 60 minutes: β of -0.27 and p-value of <0.01) and the habit of vaccination in campaigns (β of -0.54 and p-value of <0.01) were associated with lower lack of confidence in vaccines.

The deliberate delay or decision not to vaccinate their children was associated with lack of confidence in vaccines (β of 0.17 and p-value of 0.01) and greater risk perception of vaccines (β of 0.23 and p-value of 0.03), and previous experience with adverse reactions to the vaccine was associated with greater risk perception of vaccines (β of 0.33 and p-value of <0.01).

## Discussion

This is the first study to use the tool to analyze maternal vaccine hesitancy on a Likert scale with primary data from a probabilistic sample in Brazil. Only a few studies so far have addressed the refusal or voluntary delay in vaccination in the Brazil Unified Health System [4,28–31].

The study showed that mothers have a positive perception towards vaccination and trust the vaccination program. Furthermore, the following socioeconomic and demographic factors, access, use and rapport with health services and child’s vaccination characteristics are associated with more trust (and lower risk perception) from mothers towards vaccination: family income between two and five minimum wages or higher than eight minimum wages, willingness to wait longer than 60 minutes for vaccination and habit to participate in vaccination campaigns. On the other hand, poor rapports between mothers and healthcare workers, families with two or more children and deliberate decision to not vaccinate or to delay the child’s vaccination are associated with distrust and a higher risk perception of mothers towards vaccines.

In a different context of this study, Luyten; Bruyneel; Van Hoek, (2019)[38] also found similar results in a study conducted in the United Kingdom, and Domek et al., (2018) [39] in Guatemala. They identified that a large part of their sample was favorable to vaccination and confident regarding its benefits. Only a small fraction showed hesitancy. This variable (vaccine hesitancy) is complex and specific to the context, thus it may vary over time and according to location, type of vaccine and other factors, therefore other studies have proposed to assess vaccine hesitancy in different contexts [23,40–43].

The study showed that mothers with a higher family income had greater confidence in vaccines and lower perception of risk. The higher is the socioeconomic level, the lower are the chances of parents to hesitate (e.g.: they are even able to provide a booklet with all updated vaccines). This shows that higher-income families potentially present greater of access to information regarding the safety and effectiveness of vaccination and greater use/access of health services. In summary, social inequality represents a great health risk, as children born in underprivileged have a high probability of not being vaccinated [21,44–48].

Additionally, the poorer the rapport between mother and healthcare workers, the lower is the trust in the vaccine, which is supported by the findings of an Australian study that identified an association between pregnant women’s hesitancy to vaccinate their children and trust decrease in the child’s physician (p <0,0001) [49]. A good rapport between mothers and health professionals is essential to build trust, bring parents closer to vaccination services and programs, ensuring, as consequence, that the information about this subject comes from a reliable source. Extra time must be made available to interact and communicate for parents who are hesitant or abstain from vaccinating their children. In addition, repeated meetings and dialogue are essential, as well as the repetition of information about the benefits of vaccination [50]. Figueiredo et al., (2020) [22] pointed out that, among other factors, trust in healthcare workers and the information guided by them, were associated with greater chances of acceptance of the vaccine.

Nonetheless, the deliberate delay or decision not to vaccinate their children was positively associated with a lack of confidence in vaccines and the perception of risk. This behavior may be related to the fear of adverse events, the low understanding of the benefits and the perception of the severity and susceptibility to the disease, which is usually considered in the decision of taking a vaccine [51]. Besides, parents, in general, are more likely to vaccinate their children against a more serious and fatal disease than a more common one and are more hesitant about new vaccines than those that they themselves had taken during childhood [51,52].

Likewise, previous experience with adverse events to the vaccine showed a positive association with risk perception. Possibly because the potential adverse reaction following vaccination may seem more likely than the disease, arousing hesitant behavior towards vaccination. Parents whose children had experienced a suspected adverse event are significantly more likely to report greater concerns about vaccine safety [41,53], and, as a result, have more doubts about safety and greater hesitation about vaccinating, in addition, to be less favorable to the use of combined and co-administered vaccines, fearing immediate reactions [54]. These concerns, including their side effects, have been discussed in previous studies as a barrier to vaccination that may lead to hesitation [42,55–59].

Another important finding is that mothers who have other children, regardless of birth order, trust less in vaccines. Nozaki; Hachiya; Kitamura (2019) [60] showed that families with only one child had higher vaccination coverage compared to those with more than two. Previous studies also indicated that vaccine hesitancy was significantly associated with having more than one child [61–63]. A systematic review of factors associated with incomplete or delayed vaccination status in several countries also showed that the presence of other children in the family causes a loss of confidence in vaccination [21]. In Brazil, this association may be related to a couple of reasons such as the difficulty of locomotion when the mother has more than one child, and the experience with the previous child(ren) vaccine-preventable diseases or with the vaccine (the previous experience of adverse events).

Finally, mothers who are more willing to wait longer to vaccinate and who claimed to have vaccinated their children in campaigns rely more on them. These behaviors may be related to health care awareness and autonomy in decision-making, which leads mothers to be concerned about their children’s health care and to realize the importance of vaccines. Vaccination campaigns are strategies used by the health system to expand and facilitate access to immunobiological, reach those who for some reason are unable to access them and achieve a mass vaccination that aims to improve vaccination rate among children, thus preventing deaths caused by avoidable diseases [64,65]. In addition, mass campaigns in health services, and via media, can reduce the gap between rich and poor in terms of vaccination coverage, reducing vaccine hesitancy and improving equity, even though their effectiveness depends on access [66,67].

Although the present study has limitations, given its cross-sectional nature and the fact it was conducted in only one city of Brazil, the use of a probabilistic and representative sample provided a strong set of data to explore the problems related to vaccine hesitancy. Furthermore, this may be the first research identifying maternal vaccine hesitancy in Brazil using an established and validated tool, expanding the applicability of this methodology in different contexts of the country. The findings will be useful for understanding maternal behavior and providing the possibility to develop better vaccination promotion strategies.

The phenomenon of vaccine hesitancy, growing over time, saw the emergence of SARS-CoV-2, even if in a different scenario from childhood vaccines, the ideal environment for its strengthening. Questions about the rapid development of vaccines against COVID-19, the intensification of the use of social media, as individuals remain in physical distance and social isolation, and the distance from health services, strengthened and widened the reasons for refusal or delay in the vaccination [68–70].

So, a great effort is essential to implement, as quickly as possible, public policies and public health interventions to reduce hesitation, such as: i) strengthening national immunization programs; ii) strengthening health systems so that they have a basic network with free and universal access and great capillarity; iii) strengthening surveillance of post-vaccine adverse events; iv) mobilization of health professionals; v) developing new applied technologies that increase the adherence of families; vi) wider use of electronic immunization system, between others. Yet, reinforcing the presupposition that it is necessary to understand the maternal attitude that leads to refusal or delay in childhood vaccination and the importance of health professionals who make up the vaccination services. Thus, this study’s results indicate the importance of health services and their professionals, especially nurses, in strengthening strategies to reduce maternal vaccine hesitancy. It also contributes to the expansion of scientific knowledge about the factors associated with this variable.
